# Music therapy in health care practice: promise, pitfalls, and policy implications

**DOI:** 10.3389/fnhum.2026.1768102

**Published:** 2026-02-23

**Authors:** Yung-Yi Lan, Rujith Kovinthapillai, Katarzyna Wieczorowska-Tobis, Sławomir Tobis

**Affiliations:** 1 Center for Medical Education in English, Poznan University of Medical Sciences, Poznań, Poland; 2 Geriatrics Unit, Department of Palliative Medicine, Poznan University of Medical Sciences, Poznań, Poland; 3 Department of Occupational Therapy, Poznan University of Medical Sciences, Poznań, Poland

**Keywords:** health policy, interdisciplinary collaboration, music therapy, music-based interventions, neurorehabilitation, non-pharmacological interventions, person-centered care

## Abstract

Music therapy has gained recognition as a safe, effective, and person-centered intervention that bridges neuroscience, medicine, and humanities. This review synthesizes current evidence on its clinical applications, mechanisms of action, ethical complexities, and policy implications. While the strongest evidence lies in dementia care, expanding research demonstrates its effectiveness in managing a wide range of conditions, including Parkinson’s disease, stroke, acquired and traumatic brain injury (ABI/TBI), schizophrenia, autism spectrum disorder, depression, insomnia, and in palliative care. Despite its therapeutic potential, implementation is frequently hindered by methodological heterogeneity, workforce shortages, limited reimbursement, resource disparities, lack of streamlined referral mechanisms, and inadequate recognition as a standard clinical practice. In addition, ethical challenges, such as informed consent, patient autonomy, and cultural sensitivity, remain central to guiding both research and clinical practices. Integrating music therapy into mainstream health policy and practice requires standardized reporting frameworks, multidisciplinary collaboration, equitable access policies, and rigorous, long-term studies assessing the cost-effectiveness, feasibility, and patient-centered outcomes. This review concludes with actionable policy recommendations that are imperative to implementing music-based interventions for person-centered, holistic care and ensuring the sustainability of health care systems in the face of aging populations and rising prevalence of chronic illnesses.

## 1 Introduction

With a dramatic increase in the global prevalence of Alzheimer’s Disease and Alzheimer’s Disease Related Dementias (AD/ADRD) due to aging populations, healthcare systems are facing an urgent question: how to enrich patient care while reducing the risks of polypharmacy and overtreatment (Gaugler et al., 2022; Parsons, 2017). Given the limited efficacy and certain adverse drug reactions (ADRs) in dementia care, physicians and healthcare providers have turned their attention to non-pharmacologic interventions, where arts are no longer peripheral to health care (Atri, 2019). The World Health Organization’s (WHO) Health Evidence Network Synthesis Report 67 remains a foundational document, summarizing information from the academic literature spanning from 2000 to 2019, and demonstrates that artistic engagement – including music, dance, literature, and visual arts – can significantly improve the course of the condition and overall well-being across patients’ lifespan (Fancourt and Finn, 2019). Among these approaches, music therapy has shown promising results and emerged as uniquely positioned to aid individuals, especially older adults, with neurodegenerative diseases and ADRD (Lin et al., 2023).

The rationale for emphasizing music therapy is threefold. First, the rising dementia burden creates an urgency for holistic, non-pharmacological methods to support patients’ cognition and manage their psychosocial and neuropsychiatric symptoms (van der Steen et al., 2025). Second, music is widely available: it is affordable, versatile, culturally adaptable, and can be provided through various avenues, from personal to group settings, and from specialist clinics to long-term care facilities (Paraskevopoulos, 2022; Wang et al., 2025; Zhi et al., 2024). Third, music offers a unique, neurocognitive engagement. When many cognitive, executive, and communicative functions may have declined in later stages of a neurodegenerative disease, musical memory circuits are often shown to be preserved even in advanced AD, allowing music to stimulate emotions, autobiographical recalls, and communications (van der Steen et al., 2025; Jiménez-Palomares et al., 2024; Sun et al., 2024; Brancatisano et al., 2020). These characteristics of music therapy make it a perfect option for those suffering from neurodegenerative conditions.

There is a need for further investigation into both the benefits and potential pitfalls of music therapy across diverse clinical populations, particularly as health care systems encounter the dual challenges of increasing dementia prevalence and polypharmacy. This article examines current evidence on music therapy, considers mechanisms of action and ethical tensions, and outlines how health care systems might scale its use responsibly in geriatrics, palliative, and long-term care.

## 2 Conceptual framework and definitions

From a health care practice perspective, music therapy is defined as a clinical, evidence-based intervention delivered by trained professionals that utilizes music to achieve therapeutic goals across multifactorial domains: physical, emotional, cognitive, and social (Bhandarkar et al., 2024; Bradt et al., 2021). Such practice distinguishes it from non-clinical music activities, such as recreational listening, background music in care settings, or community singing. While these activities may benefit a patient’s overall well-being, they lack structured evaluation, individualized planning, and therapeutic oversight that define music therapy as a healthcare discipline (Li et al., 2021; Raglio and Gianelli, 2014).

Among clinical practices, two principal modalities of music therapy, active and receptive, are recognized. As the name suggests, active (participatory) approaches involve direct patient engagement, including singing, instrument playing, improvising, or song-writing, and are associated with stimulation of memory, communication, emotional response and motor activity (Brancatisano et al., 2020; Bhandarkar et al., 2024; Lynch et al., 2021). Receptive (listening) approaches, on the contrary, rely on guided listening, allowing for relaxation, mood regulation, or reminiscence (Raglio, 2023). Importantly, effective interventions are rarely one-size-fits-all. The ideal method and optimal outcomes depend on individualization – selecting and adapting musical content that aligns with a patient’s personal history, preferences, and clinical needs (Lynch et al., 2021; Devlin et al., 2025).

Ethical principles are fundamental to the proper implementation of music therapy, given the diverse and individualized needs of patients. Patient autonomy requires that individuals have input into whether and how music is used in their care, even in situations when cognitive impairment restricts full verbal consent (Bhandarkar et al., 2024; Raglio and Gianelli, 2014; Hackett et al., 2022). Informed consent, directly from patients when possible or from caregivers when decisional capacity is compromised, remains critical to warrant safe and proper practice (Devlin et al., 2025; Hackett et al., 2022; Thompson et al., 2025). Recognizing cultural sensitivity and respecting one’s beliefs and values ensure that the selected music resonates positively rather than offensively, acknowledging that music and songs may carry personal, cultural, and spiritual meanings that can be both therapeutic and distressing (Hennenberg et al., 2023; Ramaswamy et al., 2024; Nobakht et al., 2024). A structured, comprehensive, and well-designed conceptual framework therefore navigates the tension between the need for standardized, evidence-based approaches and individualized care tailored to each patient’s unique context.

## 3 Evidence base for music therapy in health care

### 3.1 Dementia and Alzheimer’s disease

While music therapy has been recognized as an effective intervention to treating several diseases, the strongest evidence lies in dementia care. Research has shown that music enhances patients’ cognitive performance, particularly in domains such as autobiographical memory, attention, and speech (Lin et al., 2023). Additionally, active singing interventions can evoke episodic memory and bolster executive function, while receptive, listening-based approaches help reduce anxiety and agitation, both of which present significant challenges in care (Bleibel et al., 2023). Surprisingly, regions of the brain that process music often remain relatively intact even in advanced stages of AD, allowing musical engagement to persist despite the decline of other communication pathways (Jiménez-Palomares et al., 2024; Jacobsen et al., 2015).

Beyond cognitive outcomes, music therapy demonstrates consistent efficacy in alleviating neuropsychiatric symptoms. Group-based singing and instrumental sessions have been shown to reduce behavioral disturbances such as aggression, wandering, and repetitive behaviors (Gómez-Gallego et al., 2021; Thompson et al., 2021; Dorris et al., 2021). In long-term care settings, background music played during mealtimes is associated with reductions in agitation, while structured music programs correlate with decreased fall incidence, shorter hospital stays, and less frequent use of antipsychotic medications (van der Steen et al., 2025; Sousa et al., 2021; Abeywickrama et al., 2025).

The American Occupational Therapy Association recommends music-based interventions (MBIs) for adults with AD and related neurocognitive disorders, with sessions typically lasting 10–120 min, delivered 1–5 times per week, and most effective when maintained for 1–40 weeks. Both group and individualized formats improve cognitive function, though interventions tailored to personal history and musical preferences consistently yield greater benefits in cognition and mood (Dorris et al., 2021; Smallfield et al., 2024; Leggieri et al., 2019; Pérez-Ros et al., 2019). Meta-analyses and systematic reviews confirm that active music-making and music listening, delivered individually or in groups, produce small but significant improvements in cognition and emotional well-being, with longer, more frequent sessions over several months associated with stronger outcomes (Dorris et al., 2021; Leggieri et al., 2019; Pérez-Ros et al., 2019). Overall, sustained, personalized interventions maximize the efficacy of music-based approaches for neurocognitive disorders.

Music therapy is supported by moderate-certainty evidence as a non-pharmacological intervention for managing neuropsychiatric symptoms in frail older adults with dementia. It is used for reducing depressive symptoms and behavioral problems, with a low risk of adverse effects (Lin et al., 2023; van der Steen et al., 2025; Ueda et al., 2013; Zhang et al., 2017). Both group-based and individualized interventions are feasible in institutional settings, including acute and long-term care, and can be delivered by trained staff or music therapists (Sousa et al., 2021; Abeywickrama et al., 2025; Castelino et al., 2025). While there is no direct evidence that music therapy reduces hospitalization or institutionalization, improvements in neuropsychiatric symptoms may indirectly lessen the need for acute interventions (van der Steen et al., 2025; Abeywickrama et al., 2025; Castelino et al., 2025). Evidence for reducing psychotropic drug use is limited and inconsistent, though ongoing trials are investigating this outcome further (McCreedy et al., 2022; Baroni Caramel et al., 2024). Music therapy is safe and well-tolerated, with no serious adverse effects reported, even in advanced dementia (van der Steen et al., 2025; Abeywickrama et al., 2025; Castelino et al., 2025). Additionally, it may offer economic benefits by decreasing reliance on pharmacological treatments and staff time, although robust cost-effectiveness data are lacking (Wang et al., 2025; Abeywickrama et al., 2025).

Despite these promising results, limitations persist. Many studies are short in duration, employ heterogeneous designs, and inconsistently report adverse events (Fancourt and Finn, 2019). The field thus faces a paradox: while music therapy is widely perceived as safe, rigorous evidence on potential harms such as overstimulation or distressing memories remains sparse.

### 3.2 Beyond dementia: wider clinical applications in other clinical populations

Though research has largely centered on dementia care, music therapy’s clinical utility spans across many conditions, with evidence supporting its application in stroke, Parkinson’s disease (PD), schizophrenia, autism spectrum disorder as well as other disorders. In stroke rehabilitation, receptive music therapy and other interventions have been shown to stimulate neural plasticity, support verbal memory recovery, and bolster motor rehabilitation (Sihvonen et al., 2021). Neuroimaging and clinical studies suggest that both music listening and active participation can induce neuroplastic changes in the structural and functional networks controlling language, cognitive and motor functions (Raglio et al., 2017; Kunikullaya et al., 2025; Blasi et al., 2025). Additionally, meta-analyses and systematic reviews highlight continual improvements in gait parameters, upper extremity motor function, and communication outcomes ensuing MBIs in stroke patients (Magee et al., 2017; Scataglini et al., 2025).

Among patients with PD, Rhythmic Auditory Stimulation (RAS) has been widely used as an adjunct to the conventional methods of rehabilitation. In terms of gait parameters, RAS improves velocity, balance, and stride length, and reduces falls and freezing episodes (Scataglini et al., 2025; Machado Sotomayor et al., 2021). The effects of RAS are consistent across both short- and long-term interventions, demonstrating its efficacy in further facilitating functional movement, motor fluency and improving quality of life (QOL) (Zhang et al., 2017; Thaut et al., 2019; Lindop and Skelly, 2018).

In populations with severe mental illness, such as schizophrenia, music therapy, when used adjunctively to the standard treatment, has been shown to reduce affective symptoms, including depression, anxiety, and anger, and enhance social functioning (Tseng et al., 2016; Geretsegger et al., 2017). Besides ameliorating affective symptoms, music therapy significantly alleviates patients’ negative symptoms, mood, social interest, and QOL (Jia et al., 2020; Lam et al., 2023). Although long-term, more rigorous longitudinal studies are required to completely elucidate the effects and efficacy of music therapy adjunct to standard care in patients with chronic mental illness, current findings indicate promising therapeutic value.

With promising evidence of music therapy being used in various illnesses, it has also been applied to help those with autism spectrum disorder, where structured improvisation, vocalization, and listening to music help with developing communication, initiating behaviors, social interaction, and social–emotional reciprocity (Geretsegger et al., 2014; Gassner et al., 2022). In depression, both active and receptive approaches reduce depressive symptoms and anxiety, improve mood, and improve social and occupational functioning (Gassner et al., 2022; Aalbers et al., 2017). For people suffering from insomnia and other sleep disorders, the calming effect of selected music before bed time is associated with shortened sleep onset latency, improved total sleep time, and sleep efficiency – perhaps outperforming other non-pharmacological interventions (Jespersen et al., 2022; Gou et al., 2025).

Lastly, in palliative and end-of-life care, the cathartic influence of music interventions helps reduce pain, depression, and distress, as well as bring relief, and improve sense of well-being (McConnell and Porter, 2017). Importantly, music therapy also addresses the bereavement needs of family and caregivers, allowing them to cope with loss and communicate difficult emotions (Whitford et al., 2023). Music’s ability to improve mood and provide comfort and support makes it particularly valuable in situations when curative options are no longer available, yet dignity and QOL of the patient remain paramount.

## 4 Mechanisms of action

The therapeutic effects of music arise from both neurobiological and psychosocial mechanisms. Neuroimaging evidence indicates that rhythmic stimuli can entrain neural oscillations, thereby supporting motor coordination and facilitating rehabilitation following neurological injury (Emmery et al., 2023). Melodic cues have been shown to enhance language retrieval in individuals with aphasia, while the emotional salience of familiar music engages reward and memory circuits, including the prefrontal cortex, hippocampus, and amygdala, that shape affective responses and remain active even in advanced stages of dementia (Sun et al., 2024; Blasi et al., 2025; Sihvonen et al., 2017; Dan et al., 2025). These interconnected pathways help explain the consistent effects of music on mood regulation, cognitive function, and rehabilitation across diverse clinical populations.

At the psychosocial level, music fosters social bonding and mitigates loneliness. Group singing has been shown to elevate oxytocin levels and strengthen perceived social connectedness (Fogg-Rogers et al., 2016; Bowling et al., 2022). For caregivers, music offers both respite and a shared medium for engagement as verbal communication declines (Thompson et al., 2021; Lee et al., 2022). Music has also been found to improve mood, reduce stress, and enhance concentration, efficiency, enthusiasm, and task organization in work and caregiving settings (Colin et al., 2023; Bains et al., 2024). Participatory activities such as drumming, singing, or listening to music promote relaxation and well-being among caregivers and are associated with reductions in anxiety and stress (Colin et al., 2023; Bains et al., 2024; Dingle et al., 2021). Through these mechanisms, music enhances not only individual well-being but also the quality of patient–caregiver relationships.

A distinctive advantage of music therapy in dementia care is the relative preservation of musical memory and abilities even in the advanced stages of disease. Neuroimaging studies demonstrate that brain regions critical for musical processing—including the caudal anterior cingulate cortex, ventral pre-supplementary motor area, medial prefrontal cortex, and basal ganglia, often remain functionally intact despite widespread cortical atrophy and severe impairment in other cognitive domains such as language and executive function (Jacobsen et al., 2015; Thaut et al., 2020; Slattery et al., 2019). These regions also show preserved glucose metabolism relative to areas more vulnerable to Alzheimer’s pathology, supporting the robust retention of musical memory and engagement throughout disease progression (Jacobsen et al., 2015; Thaut et al., 2020). Both procedural and semantic musical memory are relatively spared, enabling individuals with late-stage AD/ADRD to recognize and respond to familiar music and participate in MBIs even when verbal communication and higher cognitive functions are lost (Sihvonen et al., 2017; Groussard et al., 2019; Cuddy et al., 2015). This neuroanatomical and functional preservation underpins the feasibility and clinical relevance of music therapy deep into the course of neurodegenerative illness. By leveraging these enduring capacities, music therapy facilitates emotional connection, autobiographical recall, and social engagement forms of interaction often inaccessible through conventional therapeutic approaches. These mechanisms highlight why music therapy resonates so strongly in geriatric and palliative care, where it addresses neurocognitive, emotional, and social needs in an integrated manner.

## 5 Ethical complexities

**Figure 1:**
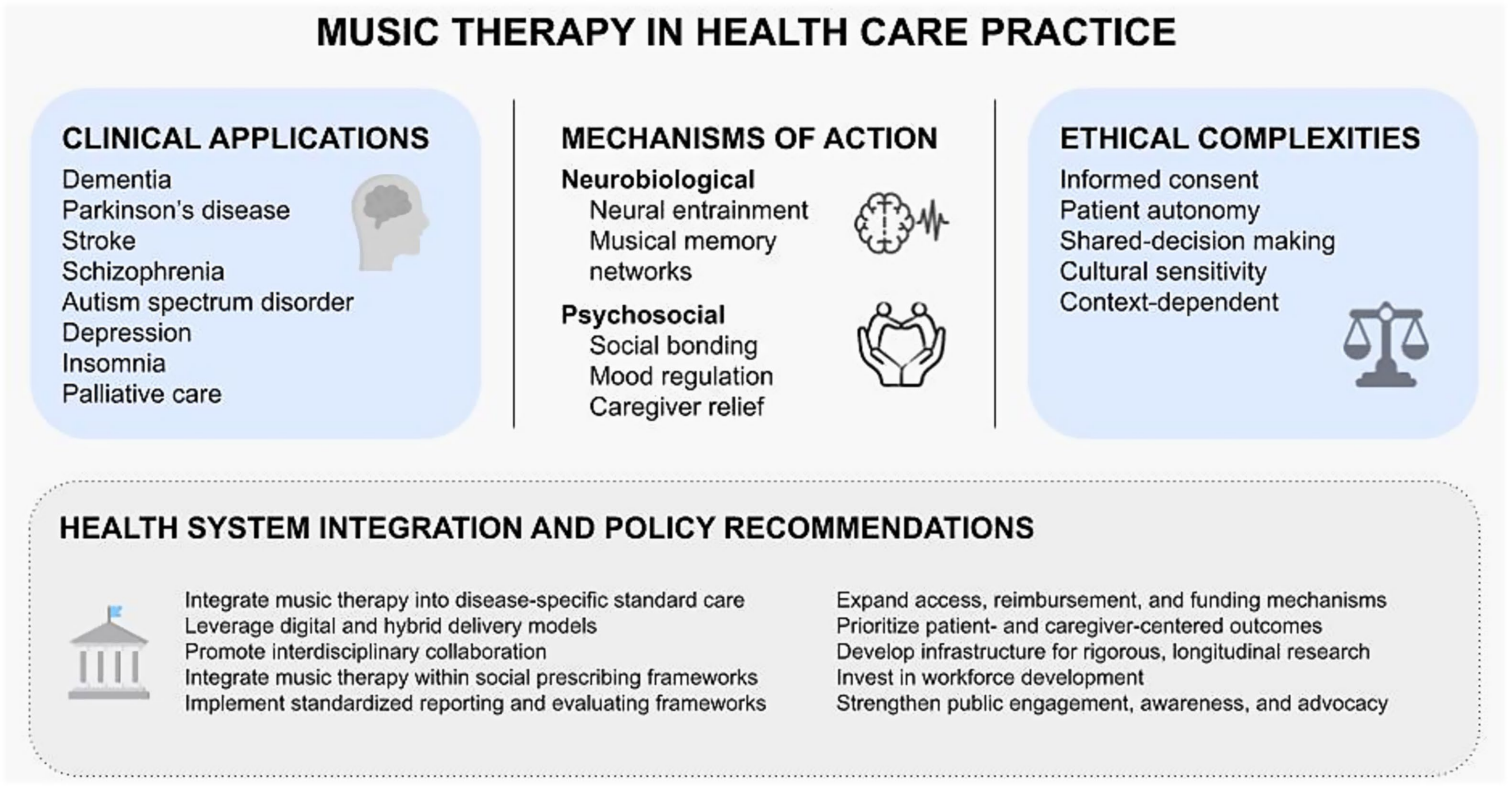
A conceptual overview of music therapy in health care practice.

Music therapy has gained traction as a complex intervention with multifaceted effects (Figure 1). Despite its therapeutic potential, music therapy is not ethically or clinically neutral. MBIs are capable of reducing pain, agitation, and neuropsychiatric symptoms, as well as enhancing memory and motor activity in dementia and other neurodegenerative conditions (Lin et al., 2023; Jiménez-Palomares et al., 2024; Koelsch and Bradt, 2025; Arnold et al., 2024). However, musical interventions can also unintentionally evoke traumatic or confusing memories, trigger overstimulation, or feel intrusive to individuals who have not provided consent or who lack cultural resonance with the selected music (Bhandarkar et al., 2024; Ramaswamy et al., 2024; Sousa et al., 2021; Sittler et al., 2021; McFerran et al., 2020; Scrine, 2021). The common assumption that music is universally soothing therefore risks oversimplifying the deeply individual and context-dependent nature of musical memory. Therapists trained to observe subtle patient responses recognize that even a seemingly benign lullaby may provoke distress in individuals for whom the music carries painful associations or in those with advanced disease stage (Sousa et al., 2021; Sittler et al., 2021). Such responses are clinically significant and warrant careful documentation, monitoring, and follow-up. This is particularly important in dementia care, where communication impairments may obscure verbal expression of discomfort, requiring heightened sensitivity to nonverbal cues (Sousa et al., 2021; Strøm and Engedal, 2021).

Ethical practice in music therapy must balance standardization, which supports evidence generation and reproducibility, with individualization, which minimizes harm. That being said, music therapy lies at the intersection of neuroscience, medicine, ethics, and health policy, as it engages neural circuits involved in emotion, memory, and motor function, and prompts inquiries regarding access and equity in health care practices (Bhandarkar et al., 2024; Sousa et al., 2021; Sittler et al., 2021; Ma and Ma, 2023). Applying core bioethical principles—autonomy, beneficence, non-maleficence, and justice—requires culturally sensitive practice, shared decision-making, and adapted consent processes, particularly when capacity fluctuates. Attention to power dynamics, including the clinician’s role in music selection, is essential to avoid imposing interventions misaligned with a patient’s values or experiences. Patient autonomy, cultural sensitivity, and informed consent must therefore remain central guiding principles in both research and clinical practice (Hackett et al., 2022; Sousa et al., 2021; Kelly et al., 2023).

## 6 Implementation and health system integration

In recent years, growing literature has supported the importance of integrating arts-based interventions within health systems, recognizing their therapeutic benefits in terms of patients’ psychological, cognitive, and QOL domains, through measurable improvements in emotional well-being, functional recovery, and overall patient satisfaction (Zhi et al., 2024; Li et al., 2021; Ma and Ma, 2023). The WHO synthesis report, a crucial work that gathers research from the past two decades, provides resounding evidence that music therapy can be effectively integrated into mainstream health policy and practice, promoting clinical outcomes and overall well-being across diverse patient populations (Fancourt and Finn, 2019). While music therapy has proved to be an essential part of many disease treatment plans, there remain various structural barriers hindering its widespread adoption. Staffing shortages represent a major constraint: the availability of trained music therapists is insufficient to meet the rising demand in dementia, serious illness, and palliative care, especially as aging populations continue to grow (Bhandarkar et al., 2024; Golden et al., 2022). Variability in therapist qualifications further complicates this issue: differences in the licensing requirements and credentialing across countries may lead to inconsistency in care and challenges in incorporating music therapy into formal health systems (Levy et al., 2025). Furthermore, a lack of standardization in the design of MBIs and methods of delivery results in substantial heterogeneity across studies and protocols, hindering reproducibility, research comparability, and slowing policy implementation (Hackett et al., 2022; Levy et al., 2025; Amano et al., 2022). These challenges are further compounded by poorly established referral systems between clinical teams and music therapists, along with disparities in accessibility, most notably in rural, underserved, or resource-constrained long-term care facilities (Golden et al., 2022; Levy et al., 2025; Amano et al., 2022).

To address these challenges, multidisciplinary care teams—in which music therapists collaborate with physicians, nurses, pharmacists, psychologists, and social workers—can adopt various models for delivering personalized care (Srolovitz et al., 2022). In-person sessions continue to remain central to music therapy practices, particularly for one-on-one sessions, individualized bedside interventions, and group-based activities. They can be tailored to patients’ specific needs and to selected musical genres or songs to better support emotional regulation, pain management, and social connectedness (Mercier et al., 2023; Cohen and Maxwell, 2020). With the advancement in technology and the growing need for telehealth during and after COVID-19, virtual music therapy or teletherapy has emerged as a more convenient, alternative method of conducting sessions, broadening access to care and allowing continuity of services for patients in remote, home-based, underserved regions, and mobility-restricted settings (Clements-Cortés et al., 2023; Kantorová et al., 2021; Agres et al., 2021). Lastly, hybrid models that combine both in-person and online sessions may provide a practical solution to accommodate patients’ work schedules, align therapeutic availability with patient needs, mitigate workforce shortages, enhance the scalability of music therapy services across diverse care settings, and optimize resource use within healthcare systems (Yu et al., 2025).

To enable a smooth integration of music therapy into the standard care, streamlined referral mechanisms, with music therapy embedded within social prescribing frameworks or electronic health record (EHR)-based pathways, can help facilitate and normalize its routine implementation in clinical practice and healthcare planning (Rodgers-Melnick et al., 2024). Simultaneously, the development of the field of music therapy in healthcare practice depends on balancing versatility with consistency. There is a need for standardized protocols to define core components of effective MBIs while allowing for individualization based on each patient’s clinical needs, medical history, and preferences. Equally important is the implementation of standardized reporting guidelines and frameworks. The *Reporting Guidelines for Music-Based Interventions* (RG-MBI), for instance, support systematic documentation of intervention content, session frequency and duration, patient feedback, therapeutic outcomes, and any adverse events (Robb et al., 2025a,b). Establishing consistent reporting practices would enhance transparency, facilitate reproducibility and comparability across studies, and enable meaningful data synthesis, thereby accelerating the translation of music therapy findings into evidence-informed policy and routine care (Robb et al., 2018; Lepping et al., 2024; Grau-Sánchez et al., 2022).

## 7 Ethical and policy considerations

With the growing application of music therapy in clinical practices, its expansion raises concerns for ethical and policy considerations that may implicate complex issues related to patient autonomy, informed consent, cultural sensitivity, professional regulation, equitable access to care, and proper integration into existing healthcare systems (Bhandarkar et al., 2024; Hackett et al., 2022; Hennenberg et al., 2023; Rajendran, 2023). Central to this matter is the need for rigorous, meticulous research design combined with transparency. Available studies from the literature vary greatly in methodology and often fail to include critical details about components of MBIs, frequency and duration, and objective outcomes, therefore impeding reproducibility and slowing research translation into actionable policy (Lepping et al., 2024). Establishing standardized frameworks that encompass intervention content, session parameters, therapist qualifications, patient responses, and safety outcomes would strengthen methodological transparency and streamline the incorporation of research findings into clinical guidelines and policy (Robb et al., 2018, 2025a; Lepping et al., 2024; Grau-Sánchez et al., 2022).

When evaluating the efficacy of music therapy in treating a patient’s condition, most current studies emphasize clinician-rated or cognitive outcomes, while overlooking aspects that are considered significant to patients and caregivers, such as QOL, psychological and emotional well-being, dignity, social connection, and caregiver burden (Lin et al., 2023; Bradt et al., 2021; McCrary et al., 2022; Köhler et al., 2020). Equally important is the integration of patient-centered and caregiver-centered measures in these assessments, thereby aligning research more closely with the lived experiences, preferences, and needs of those receiving and providing care (Hackett et al., 2022; Jodie et al., 2025). In addition, this would ensure that the widespread adoption of music therapy is implemented without sacrificing individualized care or responsiveness to diverse patient populations. The inclusion of not only patients but also caregivers in the design, evaluation, and interpretation of studies remains crucial for ethical alignment and transparency, as well as the cultivation of public trust (Hackett et al., 2022; Jodie et al., 2025).

Access to music therapy can be heavily influenced by healthcare policies, existing guidelines, and reimbursement frameworks of each country. This leads to substantial variability in the extent to which patients must pay out of pocket. Thus, there is an urgent need to address insurance coverage, reimbursement, formal recognition, funding, and access to music therapy as a part of the essential care. Music therapy has been formally recognized as a professional field in numerous Western countries, such as the United States, Canada, the United Kingdom, and Australia; yet, in many low- and middle-income countries (LMICs), there lacks standardized recognition and proper reimbursement mechanisms, aside from policy support and disparities in resource allocation (Bhandarkar et al., 2024; Golden et al., 2022). In many healthcare facilities and community settings, music therapy remains inconsistently funded, often relying on charitable support or short-term grants. To provide equitable access and ensure scalability of music therapy services across diverse care settings, government, clinicians, insurers, and healthcare payers must recognize music therapy as a reimbursable health service, especially in AD/ADRD, PD, ABI/TBI, severe mental illnesses, palliative, and long-term care where evidence is strongest (Ramaswamy et al., 2024; Magee et al., 2017; Amano et al., 2022; Yin et al., 2022). Establishing reimbursement pathways or national funding frameworks would legitimize music therapy as a part of the formal, standard practice, incentivize staff expansion, encourage training accreditation, and reduce current inequities in access to music therapy services due to geographical constraints or institutional resources (Hackett et al., 2022).

## 8 Policy recommendations

Based on the WHO synthesis report and insights taken from studies examined in this article, the authors recommend a few actionable policies:

1. **Integrate music therapy into disease-specific standard care**

Embed music therapy as an evidence-based adjunct to conventional treatment, especially for diseases such as dementia, PD, ABI/TBI, several mental illnesses, palliative care, and long-term care where evidence is the strongest, to enhance patients’ QOL and treatment outcomes.

2. **Leverage digital and hybrid delivery models**

Encourage the use of virtual music therapy and hybrid music therapy (Telehealth Music Therapy) to expand reach in rural, underserved, and mobility-limited populations while maintaining quality and personalization of care. These models do not replace the significance of in-person sessions, but act as avenues to promote equitable access and sustainable delivery.

3. **Ensure ethical and culturally sensitive practice**

Mandate ethical guidelines upholding patient autonomy, informed consent, and cultural sensitivity in both clinical practice and research to prevent harm, especially among vulnerable or cognitively impaired populations.

4. **Promote interdisciplinary collaboration**

Include music therapists within the multidisciplinary care team, including physicians, nurses, psychologists, pharmacists, and social workers, to deliver a holistic, patient-centered care that optimizes therapeutic outcomes and emphasizes enhanced communication and care coordination.

5. **Integrate music therapy within social prescribing frameworks or EHR-based pathways**

Embed music therapy referrals into social prescribing initiatives and EHR to streamline access, enhance treatment plan coordination, normalize its inclusion in routine clinical decision-making, and strengthen care continuity.

6. **Implement standardized reporting and evaluation frameworks**

Adopt standardized, structured reporting tools such as the *Reporting Guidelines for Music-based Interventions* (RG-MBI) to document intervention content, session parameters, patient feedback, therapeutic outcomes, therapist qualifications, and any adverse events. This allows for enhanced transparency, increased research reproducibility, and enriched meta-analytic synthesis.

7. **Expand access, reimbursement, and funding mechanisms**

Recognize music therapy as a formal, reimbursable health service and establish funding frameworks within national insurance and public health systems to promote equitable access and sustainable delivery.

8. **Prioritize patient- and caregiver-centered outcomes**

Encourage clinical and research evaluation frameworks to include measures of QOL, dignity, caregiver burden, psychological and emotional well-being, and social connectedness that more accurately reflect the lived experiences of both patients and caregivers.

9. **Develop research infrastructure and invest in rigorous, longitudinal research**

Fund multicenter, longitudinal studies using standardized protocols to elucidate efficacy, dose–response relationships, long-term effects, and cost-effectiveness of MBIs.

10. **Establish national accreditation, regulation and licensing standards**

Develop unified standards for education, certification, licensing, and accreditation for music therapists to ensure consistency of training, professional accountability, and quality of care across regions and care settings.

11. **Invest in workforce development to increase workforce capacity**

Address workforce shortages by investing in training programs, clinical placements, and continuing professional development of music therapists to respond to rising demand in aging and chronic care populations.

12. **Strengthen cross-sector, international collaboration and knowledge exchange**

Foster cross-sector, cross-national partnerships, linking cultural organizations with health and social care systems, to share best practices, harmonize professional standards, leverage complementary strengths, and expand the foundation for culturally sensitive MBIs.

13. **Strengthen public engagement, awareness, and advocacy**

Position MBIs as legitimate components of health promotion and clinical care through targeted public education, health promotion campaigns, and policy advocacy initiatives to enhance acceptance and uptake.

In the face of aging populations and increasing prevalence of dementia and chronic illnesses, these policy adaptations are imperative and necessary to ensure the sustainability of health care systems and uphold dignified, person-centered models of care.

## 9 Research gaps

The evidence base for music therapy is strongest in dementia, where recent systematic reviews and meta-analyses, including the 2025 Cochrane review, demonstrate moderate-certainty evidence for improvements in depressive symptoms and behavioral problems, although effects on cognition and long-term outcomes remain uncertain (van der Steen et al., 2025). However, the WHO has called for research to extend beyond dementia, recognizing the broader potential of music and other arts-based interventions across a range of health conditions and emphasizing the need for rigorous, generalizable evidence to inform policy and practice (McCrary et al., 2022; Dow et al., 2023).

A major research gap remains the limited number of large randomized controlled trials in populations other than dementia, such as autism spectrum disorder, depression, insomnia, schizophrenia, and cancer. While systematic reviews and meta-analyses suggest promising effects of music therapy on mood, social functioning, sleep quality, and overall QOL in these groups, the certainty of evidence is often low because of small sample sizes, heterogeneous intervention designs, and short follow-up periods (Bradt et al., 2021; Zhang et al., 2017; Gassner et al., 2022; Aalbers et al., 2017; Geretsegger et al., 2022; Cassola et al., 2024). Standardized outcome measures are also lacking, and adverse events are inconsistently reported, limiting the ability to assess both therapeutic benefits and potential harms systematically (Cassola et al., 2024; Golden et al., 2021).

Scalability, feasibility, and acceptability are further under-researched domains. The existing literature underscores the need for pragmatic trials and implementation studies that evaluate music therapy in real-world healthcare and community settings (Zhi et al., 2024; Dingle et al., 2021; Karkou et al., 2024). Ensuring policy relevance requires not only demonstration of clinical effectiveness but also comprehensive cost-effectiveness analyses and systematic documentation of resource requirements, workforce capacity, and reimbursement models (Zhi et al., 2024; Dow et al., 2023; Karkou et al., 2024).

Future research should therefore prioritize large, multicenter randomized controlled trials with long-term follow-up across diverse clinical populations. It should also include systematic evaluation of both benefits and harms using validated, patient-centered outcome measures, and apply implementation-science approaches to examine scalability, feasibility, and acceptability in routine care. Equally important are economic evaluations to inform sustainable policy and reimbursement frameworks, alongside broader arts-and-health research, as highlighted by recent scoping reviews, to capture the full spectrum of psychosocial and health-related outcomes (Dingle et al., 2021; Karkou et al., 2024; Fancourt and Bone, 2025).

Addressing these research priorities is essential for positioning music therapy as a mainstream modality adjunct within clinical care and for informing evidence-based policy at local, national, and international levels. Strengthening this evidence base will also help bridge the current gap between research and practice, ensuring that music therapy is implemented safely, equitably, and effectively across healthcare systems worldwide.

## 10 Future directions

Building on identified research gaps, future work should focus on conducting long-term, large, methodologically rigorous trials to generate policy-relevant data and assess music therapy’s sustained impact across diverse populations and care settings. Recent systematic reviews highlight the need for studies that move beyond short-term outcomes and small sample sizes, particularly in neurological rehabilitation, dementia, autism, and cancer care (Lin et al., 2023; van der Steen et al., 2025; Bradt et al., 2021; Sihvonen et al., 2017; Geretsegger et al., 2022). Mapping music therapy’s role across the continuum of care—from prevention (e.g., delaying cognitive decline in aging), through rehabilitation (e.g., post-stroke or chronic illness recovery), to palliative and end-of-life care—remains a key research priority, as identified in bibliometric analyses and evidence maps (Zhi et al., 2024; Ma and Ma, 2023).

Integration of patient and caregiver perspectives is essential for intervention design and outcome evaluation. Person-centered frameworks and validated patient-reported outcome measures are increasingly advocated to capture not only clinical changes but also emotional well-being, social connectedness, and caregiver burden, reflecting the lived experience of those receiving music therapy (Hackett et al., 2022). This approach is supported by recent Cochrane reviews, which emphasize the importance of patient-centered outcomes and the involvement of trained music therapists for consistent benefit (van der Steen et al., 2025; Bradt et al., 2021; Geretsegger et al., 2022).

Beyond efficacy, implementation science approaches are urgently needed to examine how music therapy can be effectively integrated within healthcare systems. Pragmatic and hybrid effectiveness–implementation studies can identify real-world barriers, facilitators, cost–utility, and workforce needs, informing scalable models for acute, community, and long-term care. Economic evaluations and policy analyses are necessary to determine cost-effectiveness and reimbursement feasibility, as highlighted in recent bibliometric studies (Zhi et al., 2024).

Advances in digital health technologies, including virtual and hybrid music therapy platforms, artificial intelligence, and wearable sensors, offer new opportunities for personalized, accessible interventions and real-time monitoring (Sihvonen et al., 2017; Chen et al., 2022). These innovations may improve reach and continuity of care for patients in remote or mobility-limited settings. However, ethical and governance frameworks must be developed to ensure data security, privacy, and transparency as these technologies evolve.

Mechanistic research should continue to explore neurobiological and psychosocial pathways underlying music’s therapeutic effects, including the use of neuroimaging, electrophysiology, and biomarkers to clarify causal mechanisms of stress regulation, neuroplasticity, and emotional modulation. Global health perspectives also warrant attention: most available evidence arises from high-income Western settings, underscoring the need for cross-cultural studies assessing feasibility, acceptability, and cost in low- and middle-income countries (Ma and Ma, 2023).

Finally, workforce development and interprofessional education should form part of future research agendas. Studies evaluating training models, accreditation standards, and interdisciplinary collaboration are essential to ensure quality, scalability, and consistency of practice. Collectively, these priorities highlight that future progress in music therapy depends not only on demonstrating efficacy, but also on achieving integration, equity, and sustainability. By bridging clinical science with implementation research, technology, ethics, and policy, music therapy can evolve from an adjunct intervention to a foundational component of person-centered healthcare.

## 11 Conclusion

Music therapy occupies a unique intersection between neuroscience, clinical medicine, and the humanities, offering an evidence-informed approach that addresses both physiological and psychosocial aspects of care. Strongest evidence exists in dementia, where music therapy improves mood and behavioral symptoms, yet growing research suggests potential across a broader spectrum of neurological, psychiatric, and palliative conditions. Despite these advances, major challenges persist: methodological variability, small sample sizes, and limited standardization hinder generalizability, while ethical and implementation barriers constrain access and equity. Future research should integrate rigorous trial design, patient- and caregiver-centered outcomes, and implementation science to evaluate scalability, feasibility, and cost effectiveness within diverse health systems. Embedding music therapy into national health policy, education, and reimbursement frameworks would support its transition from complementary practice to an essential element of person centered, evidence-based care, reaffirming the role of empathy, culture, and creativity in the modern therapeutic landscape.
